# Indocyanine Green Near-Infrared Laser Angiography Predicts Timing for the Division of a Forehead Flap

**Published:** 2012-08-31

**Authors:** Joani M. Christensen, Donald P. Baumann, Jeffrey N. Myers, Kate Buretta, Justin M. Sacks

**Affiliations:** ^a^Department of Plastic and Reconstructive Surgery, Johns Hopkins University School of Medicine, Baltimore, MD; ^b^Department of Plastic Surgery; ^c^Head and Neck Surgery, Division of Surgery, The University of Texas MD Anderson Cancer Center, Houston, TX. Justin M. Sacks, MD, is a consultant and speaker for LifeCell

## Abstract

**Introduction:** Reconstruction with flaps requiring delayed division remains common, even with increasing use of free tissue transfer. Patient quality of life and function are significantly decreased during the delay period. Delay could be minimized by developing methods to reliably determine when the flap has developed sufficient vascular supply to undergo successful division. We report the use of laser angiography to determine the appropriate time for division of a forehead flap pedicle. **Methods:** The patient who had risk factors for microvascular disease underwent near-infrared laser angiography using indocyanine green on postoperative day 21 to assess vascular perfusion of the flap. Although traditional clinical examination indicated the flap was not adequately perfused, laser angiography revealed perfusion to all areas of the flap, so the pedicle was divided. **Results:** Pedicle division was successful, with no epidermolysis or necrosis. **Conclusion:** Near-infrared laser angiography with indocyanine green can assess perfusion status of the entire flap and inform the decision to divide the flap in an objective manner.

Flap revascularization and timing of pedicle division have been targets of study dating back to the popularization of tubed pedicle flaps by Sir Harold Gillies in the early 1900s. Although tubed pedicle flaps are more of historical interest today, reconstruction with flaps that require a delay before division and inset is still common. Cross finger and thenar flaps are frequently used in hand reconstruction, and the paramedian forehead flap (PFF) remains a mainstay for reconstruction of large nasal defects. One major disadvantage of such multistaged operations is decreased patient quality of life and function in the interval between the initial procedure and pedicle inset. In the case of the PFF, wound care is required for the external pedicle, wearing glasses is precluded, and most patients remain off of work and do not go out in public until after flap division. This has led to a line of research aiming to decrease the time to revascularization and determine the minimum time to division still allowing flap survival.

Minimizing the wait before flap division is the goal, but this must be done safely, with steps taken to ensure flap viability. Studies have attempted to develop techniques to guide clinical decision-making regarding flap division timing. Many of the proposed techniques are not easily clinically applicable, cannot assess the entire flap, or require subjective interpretation. Thus, no single assessment technique has gained wide clinical acceptance. As a result, the surgeon subjectively decides when to divide the pedicle. The decision is influenced by a number of variables, including personal experience, anatomic variation, and patient age, nutritional status, and comorbidities.^1^ The decision is highly reliant upon clinical examination. Before division of the trunk, the pedicle is reversibly occluded and perfusion of the distal flap is assessed with the unaided eye. Pinprick is performed to check for bleeding. On the basis of this assessment, the surgeon divides the pedicle or leaves it in place to be reassessed at a later date. Near-infrared (NIR) laser angiography using the dye indocyanine green (ICG) approved by Food and Drug Administration can noninvasively assess perfusion of an entire flap and can guide clinical decisions regarding flap division.

## METHODS

Here we report the case of a patient receiving a paramedian forehead flap for nasal reconstruction. The patient underwent ICG laser angiography using the commercially available SPY imaging system (Novadaq, Toronto, Canada) to assess flap revascularization and inform the decision to divide the pedicle. Eight miligrams of ICG was injected intravenously followed by a 10 cc flush of normal saline.

## CASE PRESENTATION

**Figure 1:**
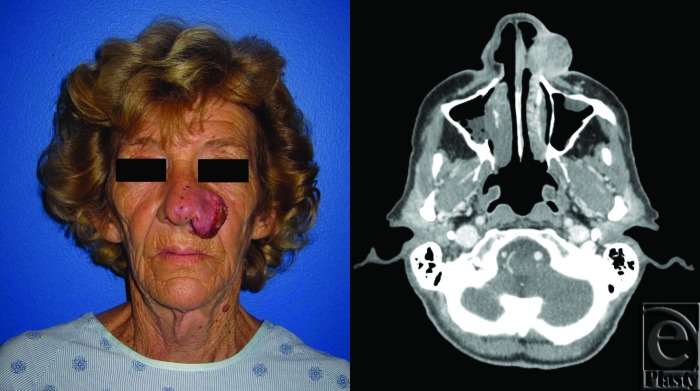
Squamous cell carcinoma of the left nose and medial cheek. Preoperative photograph (*left*). Preoperative axial computed tomographic image of invasive tumor (*right*).

**Figure 2:**
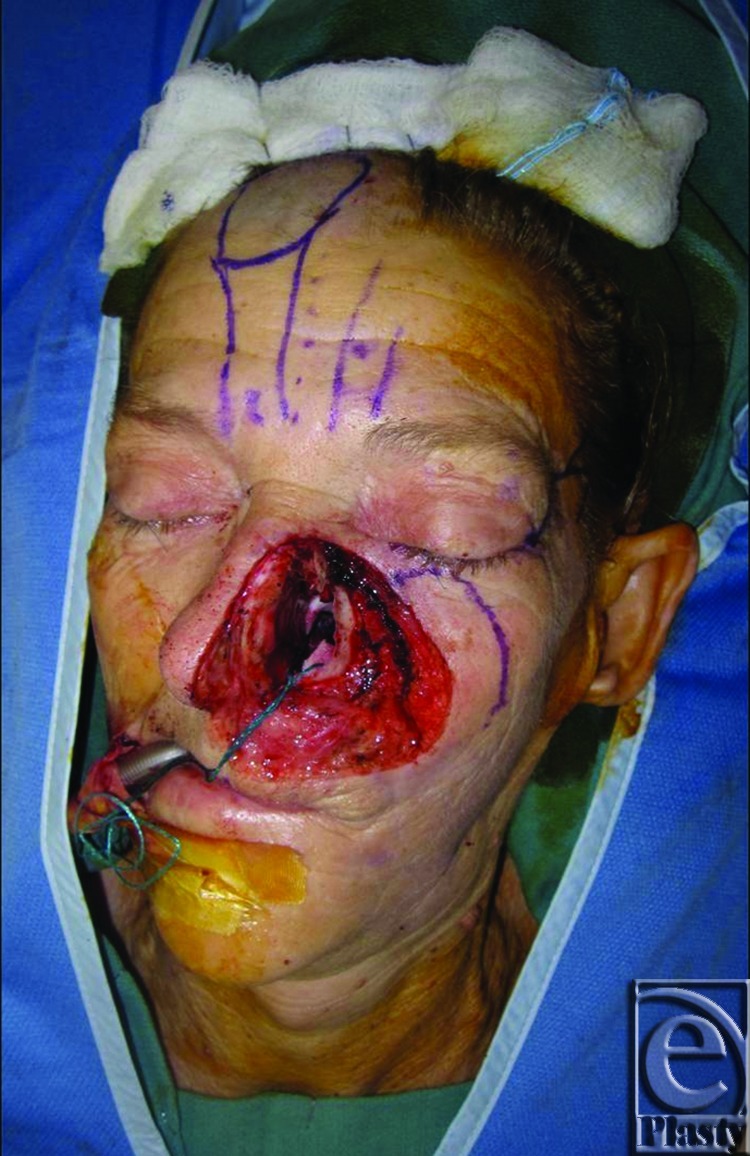
Cheek, lip, and nasal defect following wide local excision.

**Figure 3:**
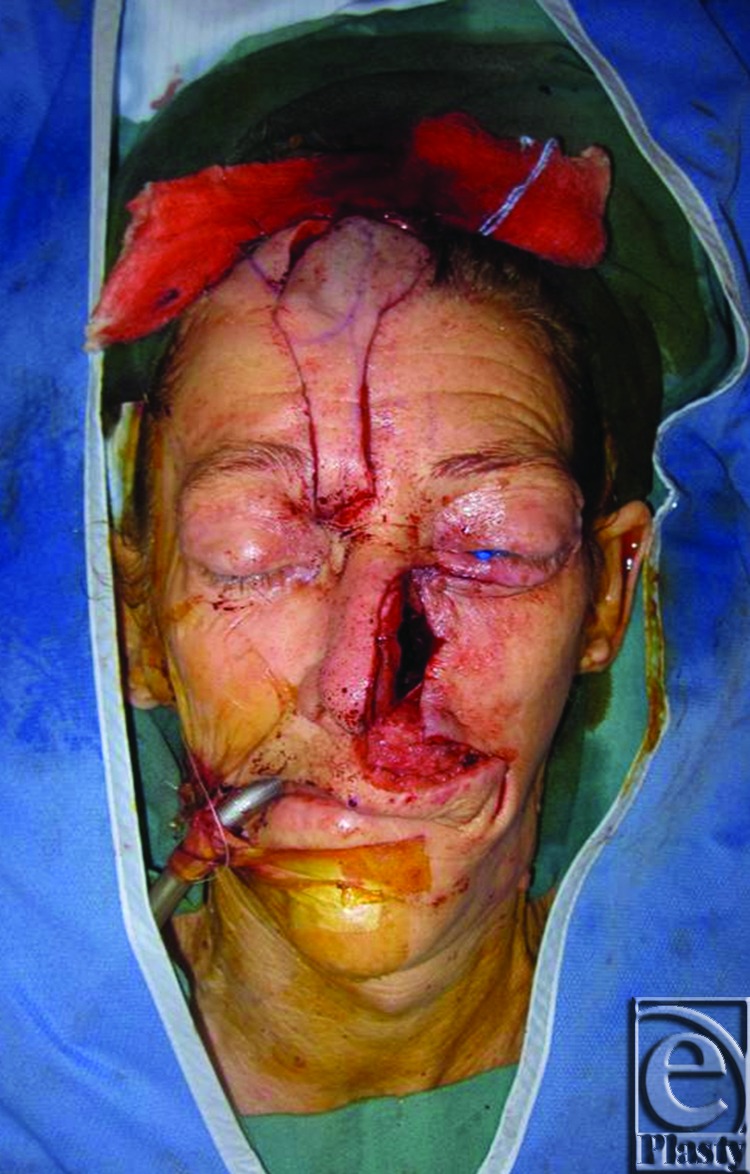
After cheek advancement, full-thickness skin graft and raising of paramedian forehead flap.

**Figure 4:**
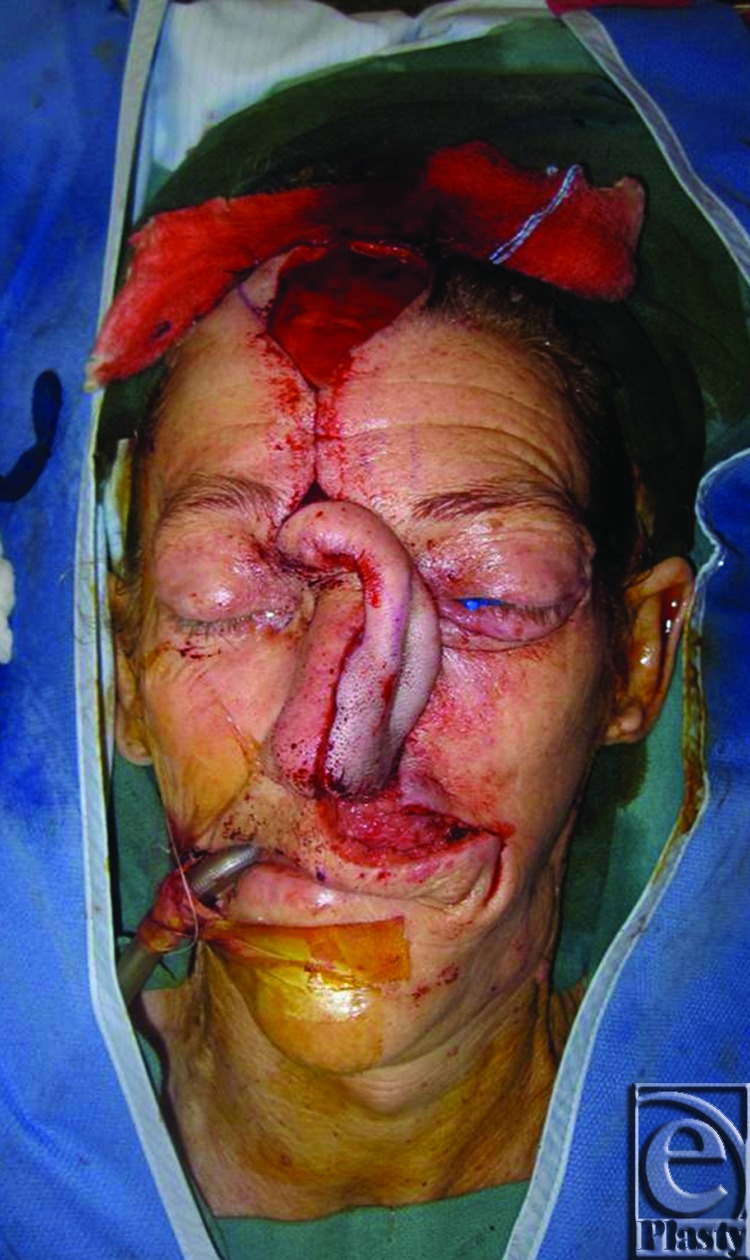
The flap was rotated into place over the defect, and the distal aspect sutured in place to the surrounding nasal and cheek skin. The inferior portion of the secondary forehead defect was closed primarily and the superior portion allowed to heal by secondary intention. The 2-cm wide pedicle was externalized.

**Figure 5:**
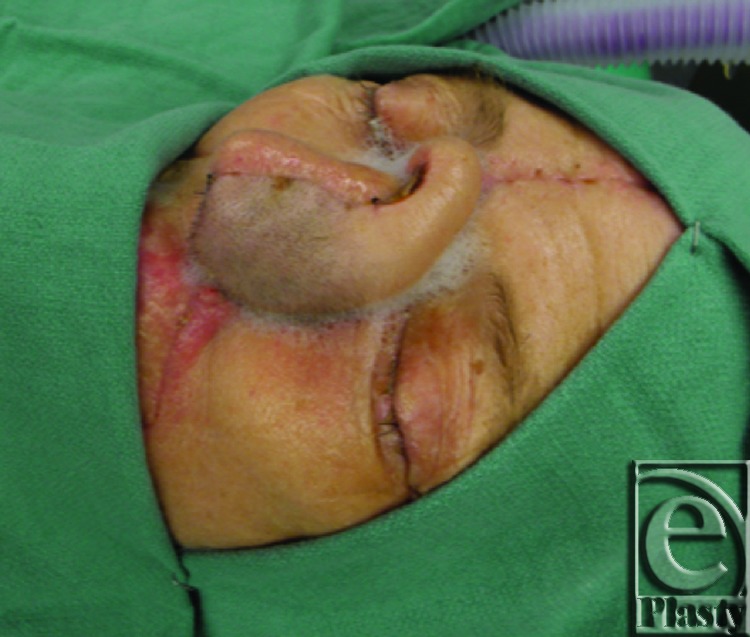
Flap inset prior to division.

**Figure 6:**
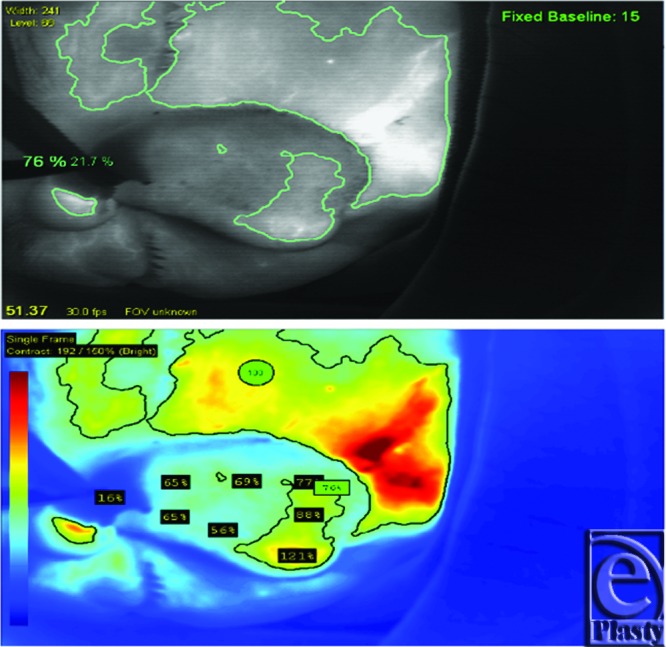
Laser angiography was performed intraoperatively at 3 weeks. Initially, there was no fluorescence in the flap, indicating successful occlusion of the pedicle *(right)*. After a few seconds, the medial aspect of the flap began to fluoresce (*middle*). The fluorescence spread over the entire flap, indicating vascular perfusion to all of the flap tissue (*left*).

The patient was a white female with a history of squamous cell carcinoma, first seen in clinic at 65 years of age. She was an active smoker with a history of hepatitis C and polycythemia vera. She presented with an invasive squamous cell carcinoma of the left nose and medial cheek (Fig 1). She underwent wide local excision of the lesion, which resulted in a full-thickness defect, encompassing the left nasal side wall, ala and soft triangle, left half of the dorsum, tip, and columella of the nose, medial left cheek, and upper lip (Fig 2). The first stage of nasal reconstruction was planned with a left cheek advancement flap and PFF based on the right supratrochlear artery, which was located using a handheld Doppler. Figure 3 illustrates the defect remaining after cheek advancement. During this initial procedure, the PFF was raised, rotated into place over the defect, and the distal aspect sutured in place to the surrounding nasal and cheek skin. The inferior portion of the secondary forehead defect was closed primarily and the superior portion allowed to heal by secondary intention. The 2-cm wide pedicle remained externalized (Fig 4). The patient was taken to the operating room on postoperative day 21 (Fig 5), and the pedicle was reversibly occluded to assess flap revascularization. Clinically, the flap did not appear to perfuse well. Upon pinprick with a 25-gauge needle, there was no bleeding. In addition, the patient had comorbidities associated with microvascular disease, which some argue warrants a delay period before division significantly longer than the standard 3 weeks. Indocyanine green laser angiography was performed with the pedicle still occluded, while in the operating room (Fig 6).

## RESULTS

**Figure 7:**
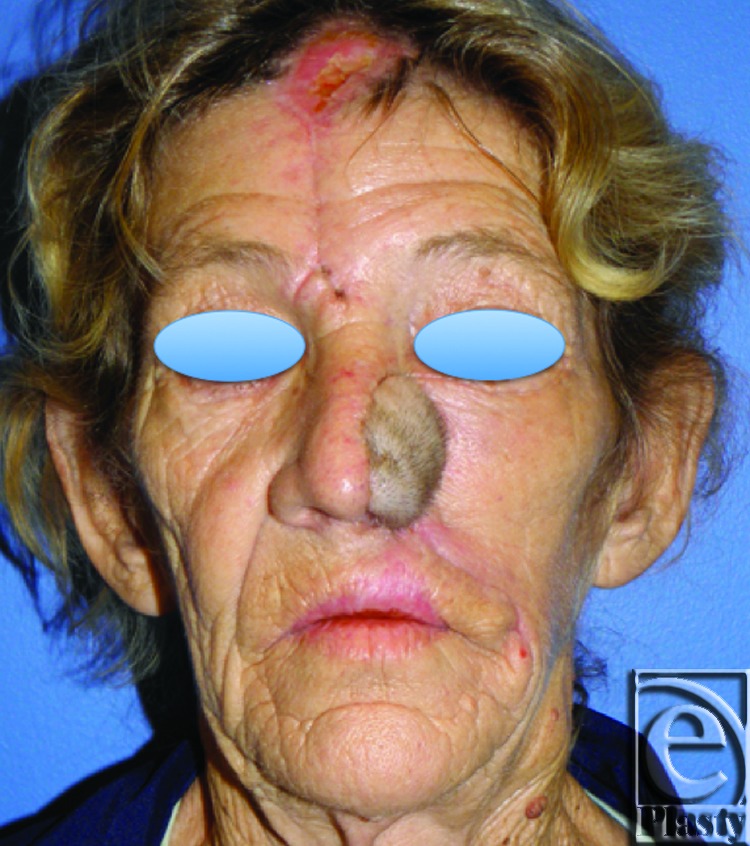
Postoperative photo, inset, and healed flap (Frontal).

**Figure 8:**
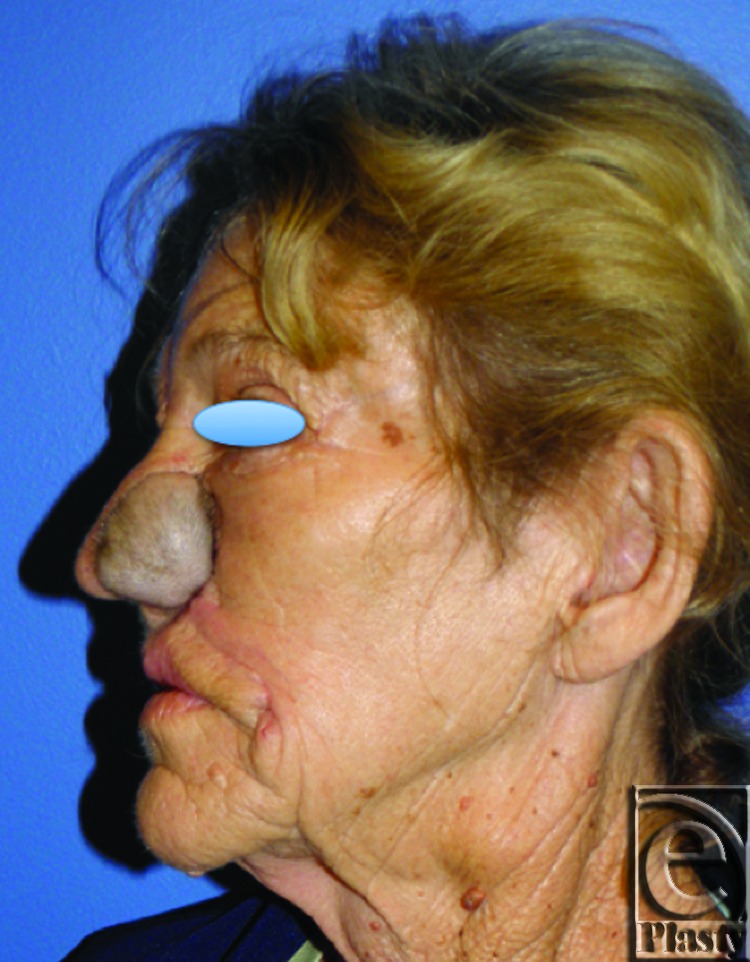
Postoperative photo, inset, and healed flap (Lateral).

Immediately following ICG injection, the proximal pedicle fluoresced, but the rest of the flap did not, as the pedicle was successfully occluded. Soon, the distal flap filled with fluorescence from the medial aspect, eventually extending over to the lateral border (see Video, Supplemental Digital Content 1, NIR laser angiography demonstrating flap filling). Because of this evidence of vascularization of the entire flap, and in spite of clinical examination and medical comorbidities, the pedicle was divided at day 21 after the initial operation. The flap remained viable, with no necrosis or epidermolysis (Figs 7 and 8).

## DISCUSSION

Two-stage reconstructions with a delay before flap division continue to be common in plastic surgery. One of the most prominent examples of this technique is the paramedian forehead flap for nasal reconstruction, which provides excellent skin matching and cosmesis. The delay before division is essential for flap survival, allowing the flap to maintain a robust blood supply while new vascular connections form with the recipient bed. However, the PFF does have significant drawbacks centered on the delay phase. After the initial operation, the external pedicle remains intact, leaving the patient with a trunk-like defect. The pedicle conventionally remains connected for 3 weeks before division and inset,^2^ with some surgeons advocating a 3-stage reconstruction including an intermediate contouring operation, requiring a 6-week delay phase.^3^^,^^4^ Recently Somoano et al conducted a telephone survey of patients who had undergone reconstruction using a PFF. Sixty-four percent of patients surveyed thought the trunk was very disfiguring and 43% reported that the externalized pedicle greatly impeded their daily activities. In patients younger than 70, the percentages rose to 100% and 63%, respectively.^5^ Wound care was categorized as somewhat to very difficult by 76% of the patients. Every patient except one responded that they rather would have had their pedicle divided after 1 week than the conventional 3 to 6 weeks.

A number of other reconstructive procedures utilize a delay to improve flap vascularity before complete separation from the donor site. The cross-finger flap is usually divided 8 to 9 days after the initial procedure.^6^ The thenar flap is divided after 10 to 14 days.^7^ Superficial circumflex iliac artery (groin) flaps used in hand reconstruction^8^ are traditionally divided after 3 weeks. Although patients receiving these flaps may feel less disfigured than those with PFFs, the delay phase in these reconstructions not only leads to discomfort and temporary inconvenience but can also cause joint contracture.

Thus, minimizing the delay between the initial and final stages of these reconstructions is of great interest. One strategy to reduce the delay phase has been to increase the rate of revascularization. Protocols using ischemic conditioning,^9^^,^^10^ hyperbaric oxygen treatment,^11^^-^13 and application of basic fibroblast growth factor^11^^,^^14^ have shown some improvement in revascularization rates. Alternatively, delay time could be reduced by reliably and efficiently determining when a flap has sufficient vascular supply to allow division. German et al began investigating the reestablishment of vascular connections within the flap in 1933 using systemic injections of toluidine blue and barium. Ten years later, Douglas and Buchholz examined a number of parameters to determine the time to division: blood pressure in the flap, hair growth rate, and rate of temperature rise after release of pedicle occlusion. They found the cumbersome temperature-return test useful for decision making in a bipedicle flap.^15^ In 1979, McGrath et al^16^ used a fluorescein dye test to determine revascularization of groin flaps for hand reconstruction and validated the test using a skin island flap in the rat. Fluorescein angiography has been used in multiple flap studies since to assess vascularity in a subjective manner. Furnas et al^9^ used perfusion fluorometry, in which a dye fluorescence index serves as an objective measure of vascularity, to successfully divide 2 flaps at 5 days after operation. Gatti et al also used fluorescein injections to assess flow and were able to divide 2 cross-leg flaps at 11 days. Although fluorescence can be objectively measured following fluorescein injection, fluorescein is not an ideal dye for this technique. It has a long half-life and diffuses out of the vasculature and into the tissues. In addition, the ultraviolet range excitation and emission spectra of fluorescein provide very minimal tissue penetration; it can only be used for visualization to the depth of the superficial dermis.^17^ More recently, laser Doppler flowmetry, which measures the Doppler shift in a laser incident on erythrocytes as a means to assess vascular flow,^18^ pulse oximetry,^19^ and infrared thermography^20^ have all been proposed as means to determine optimal time to pedicle division, but none has found wide clinical acceptance.

The NIR fluorophore ICG provides several advantages for assessing flap revascularization. Its excitation and emission spectra in the NIR range allow deeper tissue penetration than possible with fluorescein, allowing visualization to depths up to a few centimeters. In addition, ICG does not leave the vasculature when injected intravenously, and has a short half-life within the vasculature, allowing multiple injections over time. Indocyanine green binds to plasma proteins and can be used to accurately visualize vascular perfusion. Commercial laser angiography systems that quantify perfusion through quantifying fluorescence are available, allowing an objective assessment of revascularization. Such systems have found many uses in reconstructive surgery.^17^^,^^21^^-^28 Laser angiography can be performed in the clinic, requiring only intravenous access, before taking the patient to the operating room. Indocyanine green laser angiography has been applied to many aspects of plastic and reconstructive surgery already. It has been used to design optimally vascularized pedicle and perforator flaps, to assess whole-flap perfusion quickly, and to predict necrosis after any flap is inset.^23^ The SPY-Q software used in this specific case allowed easy and rapid determination of the absolute and relative levels of perfusion of target areas.

In this case example, NIR laser angiography was used to assess the vascular perfusion status of a PFF after the first stage of reconstruction in a patient who was an active smoker. Intraoperatively, clinical judgment would have forced the operative case to be aborted without the flap being divided. Intraoperative assessment of arterial inflow and venous outflow using ICG and laser angiography allowed clinical judgment to be overruled. This decreased the subjectivity in the decision to divide the pedicle and allowed the decision to be individualized to the specific patient based on quantitative visual data. This technique also greatly increases the chance that the flap will retain sufficient blood flow following pedicle division. Although imaging was carried out in the operating room in this case, it can be performed in the clinic, without sedation, before the patient is taken to the operating room. Furthermore, preoperative laser angiography can identify a perforator perfusion zone to optimally design the flap, improving the initial vascular supply to the flap after it is raised.^29^

## CONCLUSION

Near-infrared laser angiography with ICG is noninvasive, objective, and readily clinically applicable. It provides a valuable tool to assess the perfusion status of delayed flaps and can be used to determine the optimal time for division of such flaps. Minimizing the time to division while also decreasing the chance of flap necrosis will increase quality of life for patients receiving 2-stage reconstructions, such as patients undergoing nasal reconstruction with a paramedian forehead flap.
